# Bioelectricity (electromicrobiology) and sustainability

**DOI:** 10.1111/1751-7915.12834

**Published:** 2017-08-14

**Authors:** Kenneth H. Nealson

**Affiliations:** ^1^ Department of Earth Science and Biological Sciences University of Southern California 835 W. 37th Street SHS Rm 560 Los Angeles CA 90089 USA

## Abstract

Electromicrobiology is the domain of those prokaryotes able to interact with charged electrodes, using them as electron donors and/or electron acceptors. This is performed via a process called extracellular electron transport, in which outer membrane cytochromes are used to oxidize and/or reduce otherwise unavailable insoluble electron acceptors. EET‐capable bacteria can thus be used for a variety of purposes, ranging from small power sources, water reclamation, to pollution remediation and electrosynthesis. Because the study of EET‐capable bacteria is in its nascent phase, the applications are mostly in developmental stages, but the potential for significant contributions to environmental quality is high and moving forward.

## Introduction

### Electron Transport (ET) and Extracellular Electron Transport (EET)

**Figure 1:**
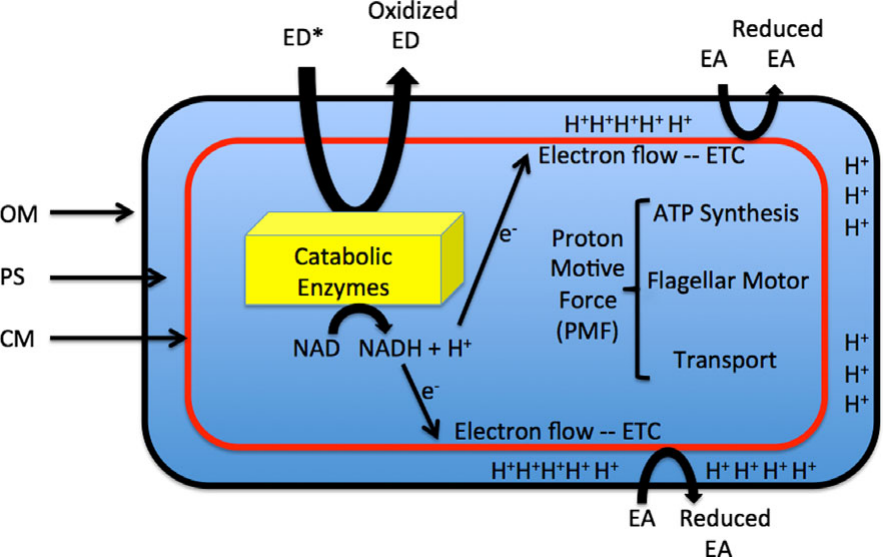
Energy generation in bacteria. Electron donors (ED) are delivered to the cytoplasm, usually by PMF‐driven transport, where catabolic enzymes are utilized to extract energy in the form of electrons, which are delivered to the cell membrane (CM) by the hydrogen carrier, NAD. These electrons flow via the electron transport chain (ETC) to soluble electron acceptors (EA). This electron flow is used to drive proton flow to the periplasmic space (PS). As the protons accumulate they establish a proton gradient called the PROTON MOTIVE FORCE (PMF) that is used to drive ATP synthesis, flagellar motility and membrane transport.

Electron flow (respiration) is the very essence of metabolic life for almost all prokaryotes (and eukaryotes via their prokaryote‐derived mitochondria and chloroplasts). The oxidative reactions by which insoluble electrons are stripped from organic or inorganic substrates and carried to the cell membrane by NAD are well‐known as are the mechanisms of electron transport down the cell membrane to an available electron acceptor (Fig. 1). During this process, the available redox energy is conserved by the pumping of soluble protons across the membrane, creating an electrochemical gradient called the proton motive force (PMF), which is used to power the synthesis of ATP, transport reactions and/or power the bacterial flagellum (Fig. 1). That is, bacteria are electrically powered organisms.

In terms of global sustainability of life, no one argues whether these properties, invented by prokaryotes are important. Sustaining life requires the resupply of nitrogen, and phosphorous, which are stripped from buried organic matter and returned to the soil or sediments or water as soluble nutrients, redistributed to the atmosphere and land via microbial redox alterations. Without these constant microbial activities, life on land would be very different from what we see now. Indeed, it is hard to imagine a sustainable planet supporting both aquatic and terrestrial life without the prokaryote‐powered reactions summarized in Table 1.

**Table 1:** Prokaryotic Contributions to a Sustainable Planet.^a^

Process	Reactants	Products	Direct functions	Indirect functions
Anoxic PS	hν, ED (H_2_, H_2_S, S^o^, Fe^2+^)	ED_ox_ PMF	ATP Synthesis Motility Transport	C‐fixation Biosynthesis N‐fixation
Oxygenic PS	hν, ED (H_2_O)	Oxygen PMF	ATP Synthesis Motility Transport	C‐fixation Biosynthesis N‐fixation
Aerobic heterotrophy	Organic C EA (O_2_)	H_2_O, CO_2_ PMF	ATP Synthesis Motility Transport	Nutrient Recycling Biosynthesis
Anaerobic heterotrophy	Organic C EA (${\text{NO}}_{3}^{-}$, ${\text{SO}}_{4}^{2-}$ CO_2_)	EA_reds_ PMF	ATP Synthesis Motility Transport	Nutrient Recycling Biosynthesis N‐fixation
Lithoautotrophy	ED_inorganic_ (H_2_, H_2_S, Fe^2+^,S^o^)	ED_ox_ PMF	ATP Synthesis Motility Transport	Nutrient Recycling Biosynthesis C‐fixation

a This list is meant only as a guide, not a comprehensive summary. It makes two points. The first is that so many crucial features of life are driven by electron flow and the formation of a proton motive force, and second, that the direct result of the electron flow is similar for all of the groups, while the contributions the organisms make to the environment are crucial and variable.

But can the arsenal of microbial metabolism be used to solve any of the present and emerging problems facing a growing human population: energy, water, waste and pollution? Here I address the properties of the ‘electric bacteria’, with the goal of distinguishing between the hype and the reality, and pointing to ways that this group of microbes, with their eclectic and electric life styles might impact our quest for sustainability.

## Who (and what) are the electric bacteria?

The idea of electric bacteria is not a new one: experimental data were first reported in 1911 by Potter (1911) who demonstrated current production by both yeast and bacteria. Several other efforts were reported by a number of workers in the mid 20th century, but as with the earlier experiments, the current production was very low, and any potential applications as power sources were not taken seriously. In recent years, the situation has begun to change. In 1988, two papers appeared nearly simultaneously, describing two different bacteria that were capable of growth on solid metal (iron or manganese) oxides as electron acceptors (Lovley and Phillips, 1988; Myers and Nealson, 1988). One of these, ultimately named *Shewanella* (Myers and Nealson, 1988) was a facultative aerobe, isolated from the oxic/anoxic interface of Oneida Lake, N.Y., where it was responsible for the rapid rates of manganese reduction seen in the lake, and shown to be able to catalyse rapid metal oxide reduction in the laboratory and to grow with solid manganese oxide as the sole electron acceptor. The other, ultimately named *Geobacter*, was an oxygen‐sensitive delta proteobacteria that was isolated from deep sediments of the Potomac River, N.Y., where it was catalysing the rapid reduction of iron oxides. (Lovley and Phillips, 1988) For almost 30 years, these two very different microbes have served as the model organisms for mechanistic studies of a process now referred to as extracellular electron transport or EET.

**Figure 2:**
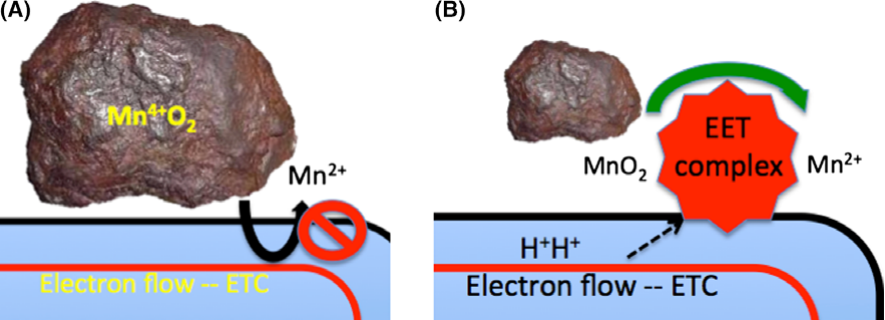
The problem with insoluble electron acceptors. Panel A demonstrates the problem that must be solved for EET to occur. Panel B represents diagrammatically the way that the problem is solved by *Shewanella* strains. It is effectively a bypass across the periplasm and through the outer membrane by a series of c‐type cytochromes called the EET complex.

It is the ability to perform EET that separates the electric bacteria from the rest of the microbial world. While almost all energy‐generating biosystems (bacteria, archaea, mitochondria and chloroplasts) operate via electron flow, they are, for the most part, designed so that electrical ‘conductors’ –the energy‐conserving membrane systems – work with soluble electron donors and acceptors, with little or no electron loss to the cell exterior. In stark contrast, EET‐capable microbes are equipped with molecular machines capable of opening new windows of metabolism – access to the world of insoluble electron donors and acceptors (Fig. 2A and B).

**Figure 3:**
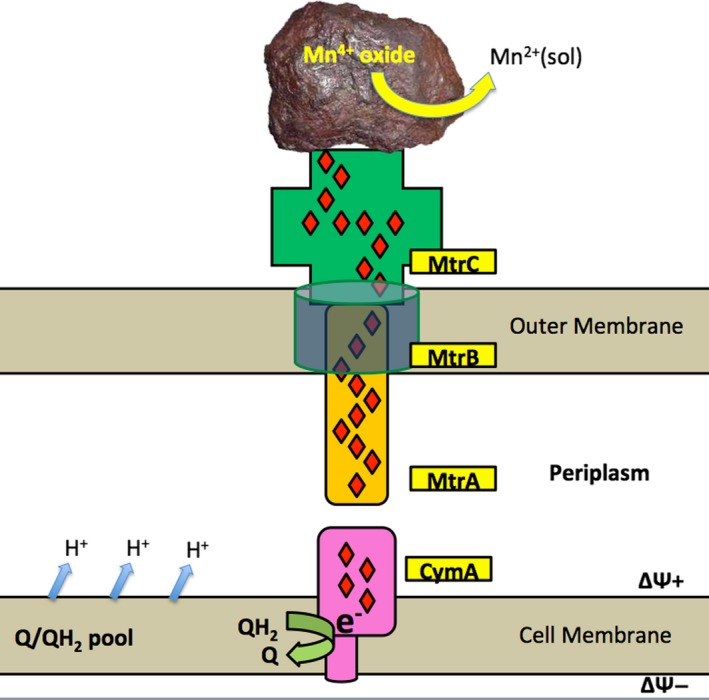
Solving the ‘EET problem’. As electrons flow down the electron transport chain, they are diverted by transfer to a tetraheme cytochrome, CymA, which carries the electrons to a decaheme cytochrome, MtrA. MtrB is a beta barrel porin‐like protein that serves as an anchor for MtrA to transfer the electrons to another decaheme protein (MtrC) located on the outer membrane, where it can interact with insoluble substrates like iron or manganese oxides.

For *Shewanella* species and strains, the mechanism by which this occurs is well understood, with a series of multiheme proteins that work together as an electron conduit (Clarke *et al*., 2011; Richardson *et al*., 2012) to move electrons from the inner membrane, across the periplasm and to the insoluble substrates at the exterior (Fig. 3). Once EET has delivered the electrons to the exterior, several variations on the theme are known in different *Shewanella* strains (El‐Naggar and Finkel, 2013), including direct reduction, reduction by exogenous and/or endogenous electron shuttling compounds (Marsili *et al*., 2008), and long‐distance reduction via cytochrome‐containing conducting membrane extensions called nanowires (Pirbadian *et al*., 2014).

For *Geobacter* species, similar multiheme c‐type cytochromes are apparently utilized, although no endogenous electron shuttles are produced, and the conducting elements are reported to be conductive pili containing no c‐type cytochromes (Malvankar *et al*., 2011). Of great interest are strains of EET‐capable microbes that contain no multiheme c‐type cytochromes, and strains of EET‐capable firmicutes (Wrighton *et al*., 2011), both of which imply that there are other EET mechanisms yet to be characterized.

The above noted model organisms were also the first microbes accused of being ‘electric bacteria’, ‘electricigens’, ‘exoelectricigens’, or any of a number of different monikers. As noted above, electrically active bacteria were not taken seriously until the discovery of EET: until the published work of Dr. Byung‐Hong Kim (Kim, 1999), in which *S. oneidensis* MR‐1 was shown to directly reduce electrodes, producing a significant level of current without the addition of electron shuttles. After this report, a flurry of activity followed in which the demonstration of good current production with high coulombic efficiency were obtained, providing impetus for research in many laboratories around the world (Logan *et al*., 2006; Lovley, 2006; Rabaey *et al*., 2007). The next development was the demonstration that microbes could also take up electrons from electrodes, and that this energy could be used for growth and/or maintenance. The realization that this process also involved multiheme c‐type cytochromes (Beckwith *et al*., 2015; Fredrickson *et al*., 2008) and that electrodes could be used to isolate such electrotrophs from many different environments. (Beckwith *et al*., 2015; Rowe *et al*., 2015).

## Contributions to sustainability?

With more than two decades of work on the model organisms, consensus has been reached that bacteria are capable of production and/or consumption of electricity, and that these processes are involved with many redox activities. But can any of these abilities be ‘captured’ for use in the name of sustainability? And if so, what are the appropriate scales of operation? Table 2 presents a brief list of potential applications, briefly discussed below, with a mind to where the opportunities lie, and where new knowledge is needed.

**Table 2:** Some potential uses and applications for Bioelectrochemical Devices

Category	Input	Advantages	Drawbacks
Energy production	Biological wastewater (WW)	Low cost nutrient and H_2_O reclamation	Low energy yield in development
via MFCs	Human WW Agri WW Industrial WW Food waste	No CH_4_ Minimal sludge N & P recovery	In development No scale‐up yet
	Air cathode for MFC H_2_O cathode for MFC	More energy Less energy	Less H_2_O recovered More H_2_O recovered
Sediment batteries	Organics in sediments	long‐life power sources	Low energy yield Difficult to deploy
Metal remediation	Metal contaminated sediments	Economical Removal of metals from environment	In development No scale‐up yet
Electrosynthesis	DC via cathode	Economical clean	In development
Academics & education	Variety of inputs Anodic or Cathodic current	Simple Easy Inexpensive	Special equipment and training may be needed

### Energy production

**Figure 4:**
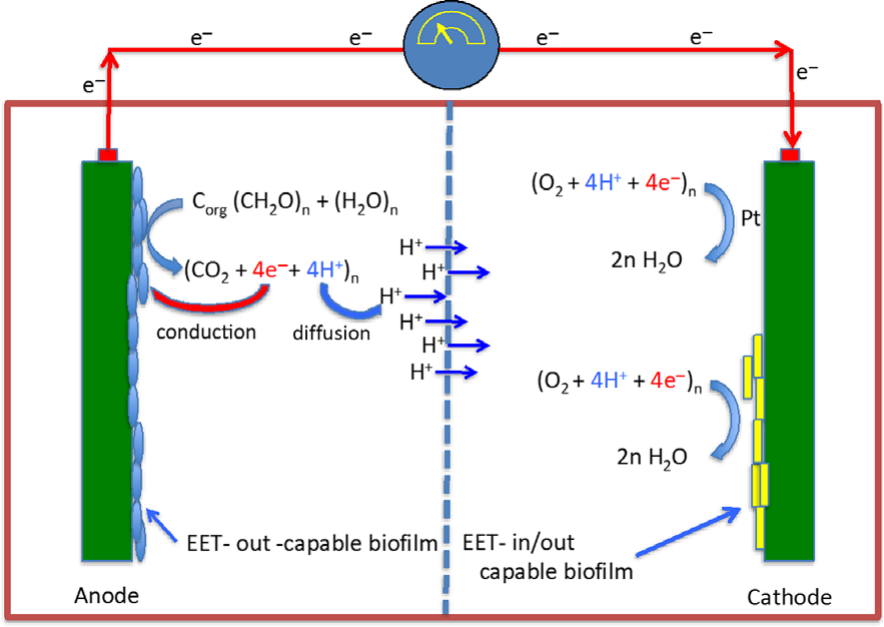
Elements of a microbial fuel cell. A ‘'standard’ microbial fuel cell consists of an anoxic anode chamber in which the only EA is the anode. A microbial biofilm is formed by EET‐capable cells that respire the organic ED, releasing soluble protons to the environment, and electrons to the anode, where they are conducted to the cathode. The protons diffuse to the cathode chamber, where they are combined with the electrons and molecular oxygen to yield water. This can be done either by a platinum catalyst or by EET‐capable microbes.

Microbial fuel cells (MFCs) operate as shown in Fig. 4, with organic materials being supplied in the anode chamber, where the electrode is adjusted to a potential the bacteria can use as an electron acceptor. Electrons flow via a conductive wire to the aerobic cathode chamber, where they combine with the diffusing protons and molecular oxygen to produce water. These devices are well‐known and have been used for energy production from organic substrates, and human, agricultural or industrial wastewater. Because they are used to breakdown biological material and produce minimal waste material and no methane, they can be regarded as renewable energy sources and as environmentally sustainable. While this is true, as noted in Table 2, the power densities and current yields are small, and the overall cost of the energy is high per unit of power produced. In the age of solar and wind energy dominance, the potential of these devices for impacting the energy portion of sustainability is imaginary, unless one is in a place with low or no light, and insufficient air movement for wind‐generation of energy.

Of course there are such places, and even with all that is said above, if uses can be designed for bioelectrochemical devices, they have the potential for being environmentally useful and running under their own power. I suggest calling these bioelectrical systems S‐BEDS – Sustainable Bio‐Electric Devices! As outlined below, one can imagine a wide variety of uses, many of which have already been tested in the laboratory and/or the field. From the point of view of environmental quality and/or human health, such devices could offer great advantages, especially in locations where power grids are absent or unreliable.

### Wastewater reclamation

Since the first demonstration of MFCs, huge strides have been made in the use of bioelectrochemical systems for wastewater remediation. These advances involve the movement from pure cultures to mixed communities of microbes that are robust to changes in substrate input (Ishii *et al*., 2013, 2015), and in general, produce higher power yields than can be achieved with pure cultures. When an air cathode is used instead of an immersed cathode, the higher concentration of oxygen allows the reaction to proceed faster and with a higher energy yield. However, if a water cathode is used, the pure water produced in the cathode chamber is collected for potential re‐use. Demonstrations of such systems have been attempted with industrial and municipal waste streams, but to date no large‐scale demonstrations have been acomplished. In the latter case, efficient removal of BOD and COD was seen, and little or no sewage sludge was produced (Ishii *et al*., 2011), but scaling the laboratory systems to municipal scale is ‘work in progress’. Such systems are of great interest with regard to human health, as they could allow water reclamation to occur where power grids are not available, thus substituting for unhealthy methods of sewage disposal. In such a situation, a small power yield could pay a large sustainability dividend in terms of energy, water and waste (i.e. environmental quality).

### Sediment batteries

Some of the most common manifestations of bioelectrical devices are the so‐called mud batteries in which the anode electrode is simply placed in an organic‐rich sediment and connected to the cathode in the overlying aerobic water. When EET‐capable bacteria are added, they begin to respire the electrode and degrade the organics in the sediment and current is produced. This is a revealing experiment for young scientists, and the beginning of understanding of what we call sediment batteries – units capable of generating small amounts of current that can be used to power sensing devices or other low power consuming units on the dark ocean floor or other places where the sun or the wind are not available (Nielsen *et al*., 2008; Reimers *et al*., 2006).

### Pollution remediation

One of the defining features of metals is their ability to be easily oxidized or reduced: Mn^4+^ oxides are solids, and when reduced become soluble salts of Mn^2+^ (e.g., MnCl_2_), while oxidized forms of U or Cr are toxic in large part because of their high solubility, and when reduced, they become insoluble metal hydroxides. Thus, if one designs an S‐BED unit with this in mind and provides it with the proper bacteria, it is possible to use such systems to reduce soluble uranium or chromium to their insoluble forms, capturing them in the cathode chamber and efficiently removing them from the environment (Hsu *et al*., 2012).

### Electrobiosynthesis

When it was discovered that bacteria could be maintained on the cathode, using electricity as their source of energy, the field of electrobiosynthesis was born – the notion of using specialized bacteria capable of electron uptake via EET for the synthesis of specific products (Rabaey *et al*., 2011; Rabaey and Rozendal, 2010). This application is in its nascent stages, but offers immense potential in terms sustainable product production – producing valuable chemical products with solar power as the feedstock.

## Conclusion

The world of electromicrobiology is a growing area of microbiology and of biology in general, and with a constant stream of new developments, there is an expectation of continued growth. There are many more potential applications now being studied, including corrosion inhibition, biofilm formation, biosensors and others. Here I have tried to focus on developments that might impact our needs in terms of sustainable systems. This area of microbiology, which was unknown 30 years ago, may provide some of the most exciting and useful tools in the quest for our ‘sustainable future’.

## Conflicts of interest

None declared.
